# Synthesis and characterizations of highly luminescent 5-isopropoxybenzo[*rst*]pentaphene

**DOI:** 10.3762/bjoc.21.19

**Published:** 2025-02-04

**Authors:** Islam S Marae, Jingyun Tan, Rengo Yoshioka, Zakaria Ziadi, Eugene Khaskin, Serhii Vasylevskyi, Ryota Kabe, Xiushang Xu, Akimitsu Narita

**Affiliations:** 1 Organic and Carbon Nanomaterials Unit, Okinawa Institute of Science and Technology Graduate University, 1919-1 Tancha, Onna-son, Kunigami-gun, Okinawa 904-0495, Japan; 2 Organic Optoelectronics Unit, Okinawa Institute of Science and Technology Graduate University, 1919-1 Tancha, Onna-son, Kunigami-gun, Okinawa 904-0495, Japan; 3 Science and Technology Group, Okinawa Institute of Science and Technology Graduate University, 1919-1 Tancha, Onna-son, Kunigami-gun, Okinawa 904-0495, Japan; 4 X-ray diffraction facility, Department of Chemistry, 100 E. 24th Street, The University of Texas at Austin, Austin, TX 78712, USA

**Keywords:** benzo[*rst*]pentaphene, intramolecular charge transfer, nanocrystals, photoluminescence, polycyclic aromatic hydrocarbon

## Abstract

A benzo[*rst*]pentaphene (BPP) substituted by an isopropoxy group (BPP-OiPr) was synthesized in a facile manner. Its photophysical properties were investigated by UV–vis absorption and fluorescence spectroscopy in compassion to pristine BPP and its oxidation product, benzo[*rst*]pentaphene-5,8-dione (BPP-dione). BPP-OiPr exhibited a significantly enhanced photoluminescence quantum yield (PLQY), reaching 73% in comparison to pristine BPP (13%). BPP-dione, when compared to the parent BPP, also displayed improved absorption and emission from the first excited singlet (S_1_) state with a PLQY of 62% and an intramolecular charge-transfer character. The rod-like nano- to microcrystals as well as longer wires of these BPPs were also revealed by scanning electron microscopy. The intriguing optical properties of BPP and its derivatives may lead to their application as fluorophores.

## Introduction

Polycyclic aromatic hydrocarbons (PAHs) have attracted increasing attention in view of their fascinating optical and electronic properties, which strongly depend on their size, shape, and edge structures, e.g., armchair and zigzag [1–6]. Benzo[*rst*]pentaphene (BPP) is an intriguing PAH with a combination of zigzag and armchair edges, which may serve as a key building block for obtaining multifunctional organic materials [7]. Since the initial synthesis of BPP by Scholl and Neumann [8], simplified synthetic methods for BPP have been reported over the past decades [9–14]. Recently, a facile access to BPP was reported by Amsharov and co-workers through the so-called "dehydrative π-extension (DPEX)” reaction [12]. However, functionalized derivatives of BPP have scarcely been explored in comparison to the extensive studies on the derivatives of other PAHs, such as pyrene [15–17], perylene [18–19], and coronene [20]. Besides their limited accessibility in the past, the lack of attention to BPP can presumably be ascribed to its low photoluminescence quantum yield (PLQY). We recently reported a PLQY of 13% for pristine BPP and revealed a symmetry-forbidden nature of its first excited singlet (S_1_) state [21]. Notably, a dimer of BPP, 5,5'-bibenzo[*rst*]pentaphene (BBPP), exhibited an enhanced PLQY of 44% through intensity borrowing from its bright S_2_ state as well as intriguing symmetry-breaking charge transfer between two BPP units. Moreover, the substitution of BPP with two electron-donating bis(methoxyphenyl)amino groups further improved the PLQY to 73%, displaying a mixed excitonic and charge-transfer character [22]. Additionally, the functionalization of BPP with two methyl benzoate groups enabled the development of hole-selective contact, which was applicable in significantly improving the stability of inverted perovskite solar cells [23]. On the other hand, benzo[*rst*]pentaphene-5,8-dione (BPP-dione) is known as an oxidation product of BPP [24–25], but to the best of our knowledge, the detailed optical properties of this BPP derivative have not been previously described in the literature.

**Scheme 1:**
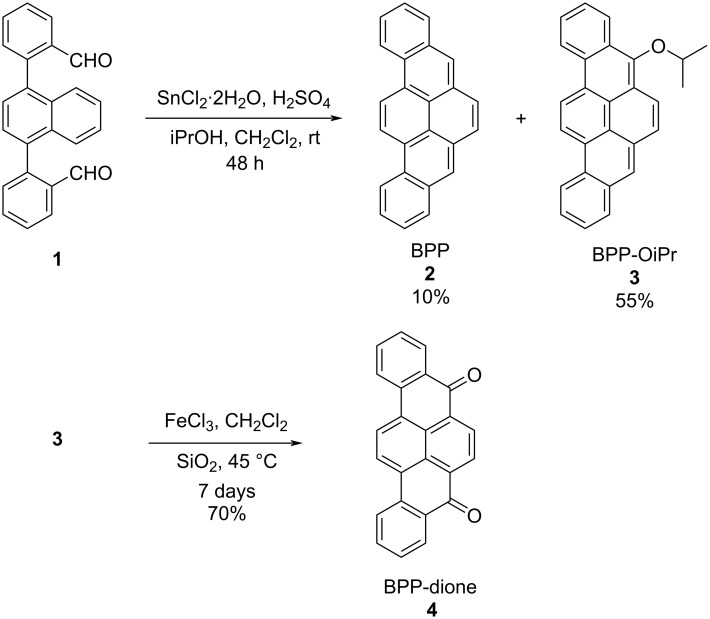
Synthesis of BPP-OiPr **3** and BPP-dione **4**.

During our attempt to scale up the preparation of BPP **2** through the "DPEX" reaction, we unexpectedly obtained a 5-isopropoxy-substituted derivative of BPP (BPP-OiPr **3**) (Scheme 1), whose structure was proven by NMR, mass spectrometry, and X-ray crystallography. In this work, we optimized the reaction conditions to selectively obtain BPP-OiPr **3** in 55% yield from dialdehyde **1**. Additionally, oxidation of BPP-OiPr **3** provided BPP-dione **4** in 70% yield. The photophysical properties of BPP-OiPr **3** and BPP-dione **4** were carefully studied, examining the solvent-polarity dependence of their optical spectra, in comparison with parent BPP **2**. Notably, both BPP-OiPr **3** and BPP-dione **4** displayed enhanced PLQYs while a significant solvent-polarity dependence of the emission was observed only for the latter, suggesting the photoinduced intramolecular charge-transfer character of **4**. Moreover, BPPs **2**–**4** formed intriguing rod-like nano- to microcrystals and/or longer wires, which were visualized by scanning electron microscopy (SEM).

## Results and Discussion

BPP **2** could be prepared by the “DPEX” reaction [12] in 60% yield on a 0.1 g scale from dialdehyde **1** with a concentration of 0.60 mM (Table 1, entry 1). However, the yield of BPP decreased to 40% when the amount of **1** was increased to 1.0 g with a concentration of 2.6 mM (Table 1, entry 2). In our attempt to improve the yield of BPP **2**, we decreased the equivalent of SnCl_2_·2H_2_O and increased the volume ratio of iPrOH, which unexpectedly provided BPP-OiPr **3** as a byproduct in 7% yield along with BPP **2** in 55% yield (Table 1, entry 3). Further optimization of the reaction conditions by modifying the equivalent of SnCl_2_·2H_2_O and volume ratios iPrOH and H_2_SO_4_ afforded BPP-OiPr **3** in 55% yield (Table 1, entries 4 and 5). Additionally, the oxidation of BPP-OiPr **3** using ferric chloride (FeCl_3_) gave BPP-dione **4** in 70% yield. The chemical structures of BPP-OiPr **3** and BPP-dione **4** were characterized by ^1^H and ^13^C NMR spectroscopy as well as mass spectrometry (see Supporting Information File 1, Figures S8–S11).

**Table 1:** Reaction conditions for the synthesis of BPP **2** and BPP-OiPr **3**.

Entry	Concentration of **1** (mM)	SnCl_2_·2H_2_O (equiv)	iPrOH (vol %)	Concentrated H_2_SO_4_ (vol %)	Time (h)	Yields of **2**/**3** (%)^a^

1^b^	0.6	40	2.5	5.0	18	60/–
2^c^	2.6	40	2.5	5.0	24	40/–
3^c^	5.1	20	4.0	5.0	48	55/7
4^c^	6.0	20	10	5.0	72	29/37
5^c^	4.8	30	14	6.0	48	10/55

^a^Isolated yields. ^b^Amount of **1**: 0.10 g. ^c^Amount of **1**: 1.0 g.

**Figure 1:**
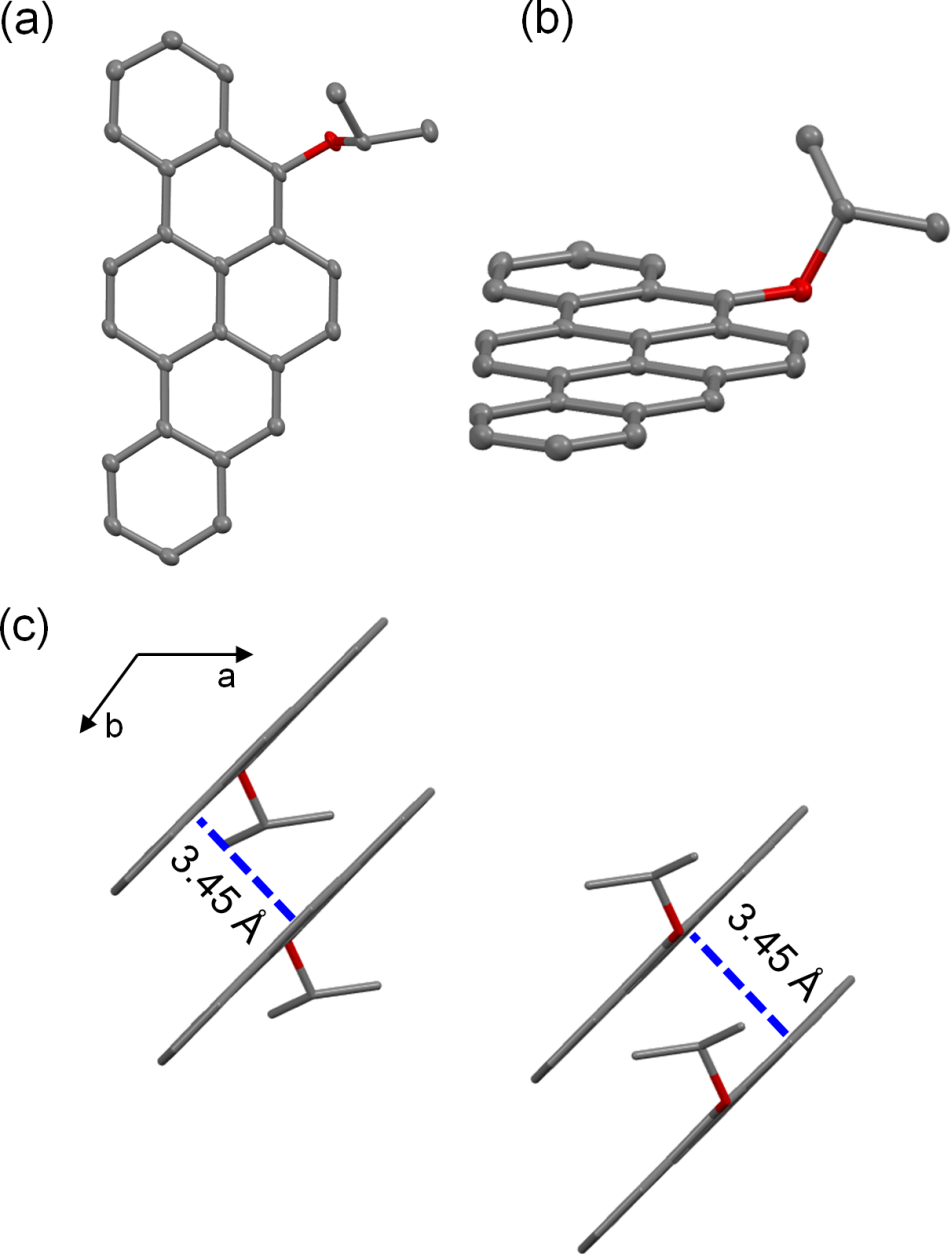
Single crystal structure of BPP-OiPr **3**: a) top view, b) side view (thermal ellipsoids shown at 50% probability), and c) molecular packing of **3** in a unit cell. All the hydrogen atoms are omitted for clarity.

A single crystal of BPP-OiPr **3** suitable for X-ray diffraction analysis was obtained by slow evaporation of a diethyl ether/*n*-hexane solution, enabling its unambiguous structural determination by single-crystal X-ray diffraction (Figure 1). The planar BPP core and the isopropyloxy group on the zigzag edge are clearly visualized (Figure 1a and b). In a unit cell consisting of four molecules, every two of them are stacked with the plane-to-plane distance of 3.45 Å (Figure 1c), displaying a lamellar π–π stacking motif in the overall packing structure (Figure S1 in Supporting Information File 1) [26–29]. The X-ray structure is well consistent with a model optimized by density functional theory (DFT) calculations (Figure S2 in Supporting Information File 1).

**Figure 2:**
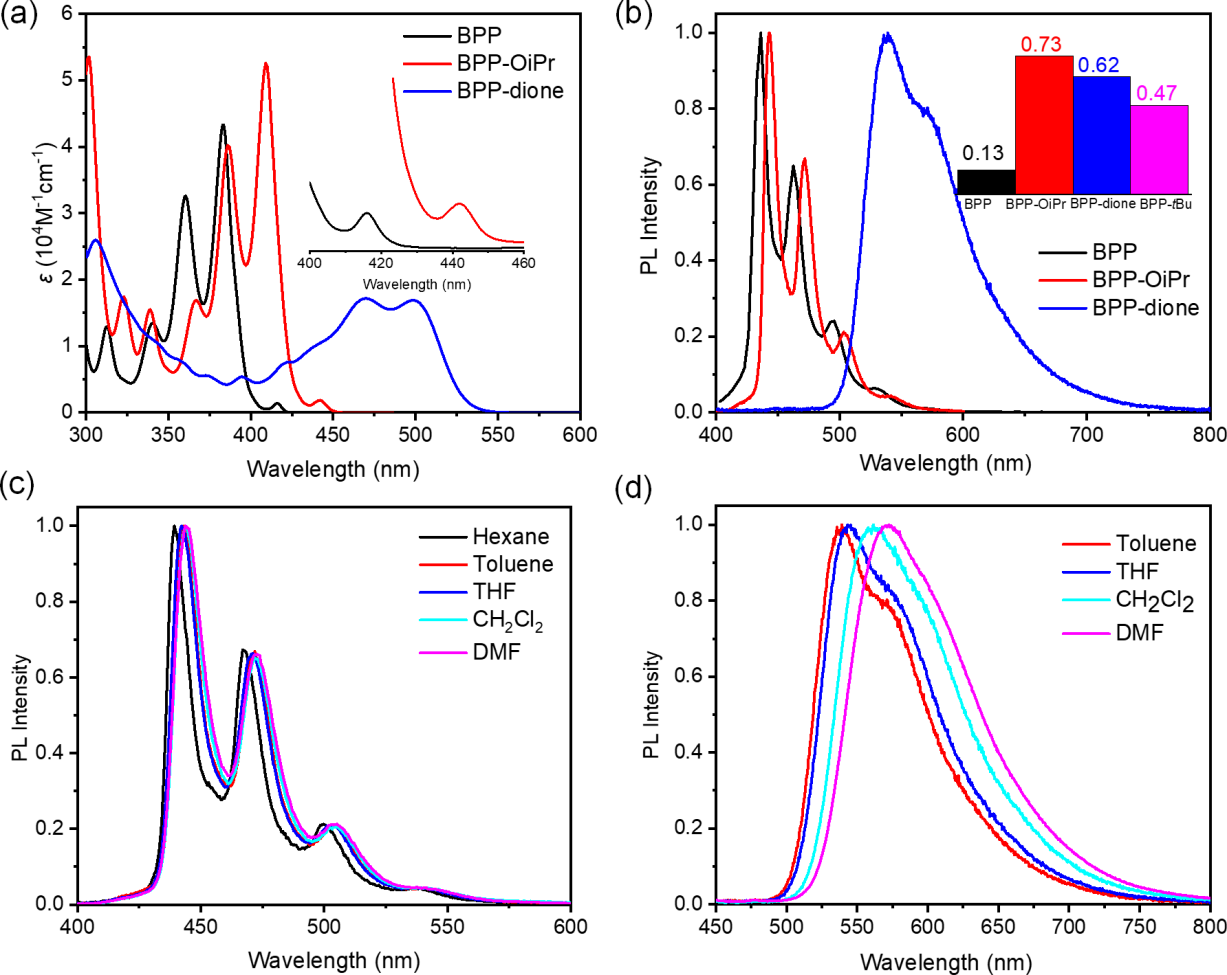
a) UV–vis absorption spectra of BPP **2**, BPP-OiPr **3**, and BPP-dione **4** measured in toluene. Inset: magnified spectra of **2** and **3** for the better visualization of the longest-wavelength peaks, b) normalized PL spectra of BPP **2**, BPP-OiPr **3**, and BPP-dione **4** measured in toluene with a 360 nm excitation. Inset: absolute PLQY of BPP **2** (excitation at 360 nm), BPP-OiPr **3** (excitation at 380 nm), BPP-dione **4** (excitation at 470 nm) in toluene, and BPP-*t-*Bu (excitation at 360 nm) [22], c) normalized PL spectra of BPP-OiPr **3** measured in hexane, toluene, tetrahydrofuran (THF), dichloromethane (CH_2_Cl_2_), and DMF, and d) normalized PL spectra of BPP-dione **4** measured in toluene, THF, CH_2_Cl_2_, and DMF.

The optoelectronic properties of BPP-OiPr **3** and BPP-dione **4** were initially investigated by UV–vis absorption spectroscopy in comparison with BPP **2** (Figure 2a). BPP **2** and BPP-OiPr **3** displayed similar and well-structured absorption peaks clearly showing the vibronic progressions. BPP-OiPr **3** also exhibited a small peak located at 442 nm with the molar extinction coefficient (ε) of 1800 M^−1^ cm^−1^, which was similar to the previous observations of the dark S_1_ states for BPP **2** and its mesityl- and *tert*-butyl-substituted derivatives [21–22]. In comparison to the absorption spectrum of BPP **2**, this lowest-energy absorption band of BPP-OiPr **3** was red-shifted by ≈26 nm, which marked the inductive and resonance effects of the electron-donating isopropyloxy group with lone pairs of electrons, raising the HOMO level (see Table S6 in Supporting Information File 1). BPP-dione **4** exhibited a broad absorption extending to 540 nm with a peak at 305 nm and featureless maxima at 469 and 497 nm, which were in line with the results of time-dependent DFT (TD-DFT) calculations at the M062X/6-311G(d,p) level of theory (Supporting Information File 1, Table S2). The longest-wavelength absorption maximum (S_0_ → S_1_), attributed to the HOMO → LUMO transition, was calculated to be at 432 nm (*f* = 0.5674) for **4**. Compared to the S_1_ states in BPP **2** (ε = 1200 M^−1^ cm^−1^) and BPP-OiPr **3** (ε = 1800 M^−1^ cm^−1^), corresponding to forbidden transitions as previously discussed for other BPP derivatives [21–22], the strikingly enhanced molar extinction coefficient observed for the lowest-energy band of BPP-dione **4** (ε = 17000 M^−1^ cm^−1^) indicates that the optical transition to the S_1_ state becomes allowed by this oxidation.

BPP **2** and BPP-OiPr **3** exhibited similar emission spectra in toluene with maxima at 436 and 443 nm, respectively, with well-defined vibronic structures (Figure 2b). Notably, the absolute PLQY of BPP-OiPr **3** was measured to be 0.73, demonstrating a remarkable enhancement of the photoluminescence from BPP **2** (PLQY: 0.13) and *tert*-butyl-substituted BPP (BPP-*t-*Bu, PLQY: 0.47) (Figure 2b) [21–22]. Considering that two *tert*-butyl groups should more effectively hinder the aggregation than one isopropyl group, we tentatively attribute the enhanced PLQY of **3** to the reduced molecular symmetry, which can relax the selection rule and allow more radiative transitions.

On the other hand, a broad and featureless PL spectrum with the maximum at 538 nm was observed for BPP-dione **4** in toluene with a high PLQY of 0.62, which is again significantly enhanced from that of BPP **2**. To gain further insight into the photophysical properties of BPP-OiPr **3** and BPP-dione **4**, their absorption and emission spectra were next measured in different solvents (Figure 2c and d and Supporting Information File 1, Figures S4 and S5). For BPP-OiPr **3** the well-defined vibronic structures were observed without showing any significant solvent-polarity dependence (Figure 2c). In contrast, BPP-dione **4** displayed a considerable redshift of the emission maximum from 538 nm in toluene to 572 nm in dimethylformamide (DMF) along with disappearance of the shoulder peak with increasing solvent polarity (Figure 2d). The UV–vis absorption spectra of **4** in different solvents also showed significant differences (Figure S5 in Supporting Information File 1), indicating an intramolecular charge-transfer character both in its ground and excited states [30–31].

DFT calculations were performed to understand the effects of the substituents on the frontier orbitals. As shown in Table S6 and Figure S1 in Supporting Information File 1, the highest occupied molecular orbital (HOMO) and the lowest unoccupied molecular orbital (LUMO) of BPP-OiPr **3** were calculated to be at −5.15 and −2.00 eV, respectively, with a slightly smaller HOMO–LUMO gap of 3.15 eV compared to that of BPP **2** (3.24 eV). BPP-dione **4** was revealed to have lower HOMO (−6.18 eV) and LUMO (−3.31 eV) and an even smaller HOMO–LUMO gap of 2.87 eV in agreement with the experimental optical spectra.

**Figure 3:**
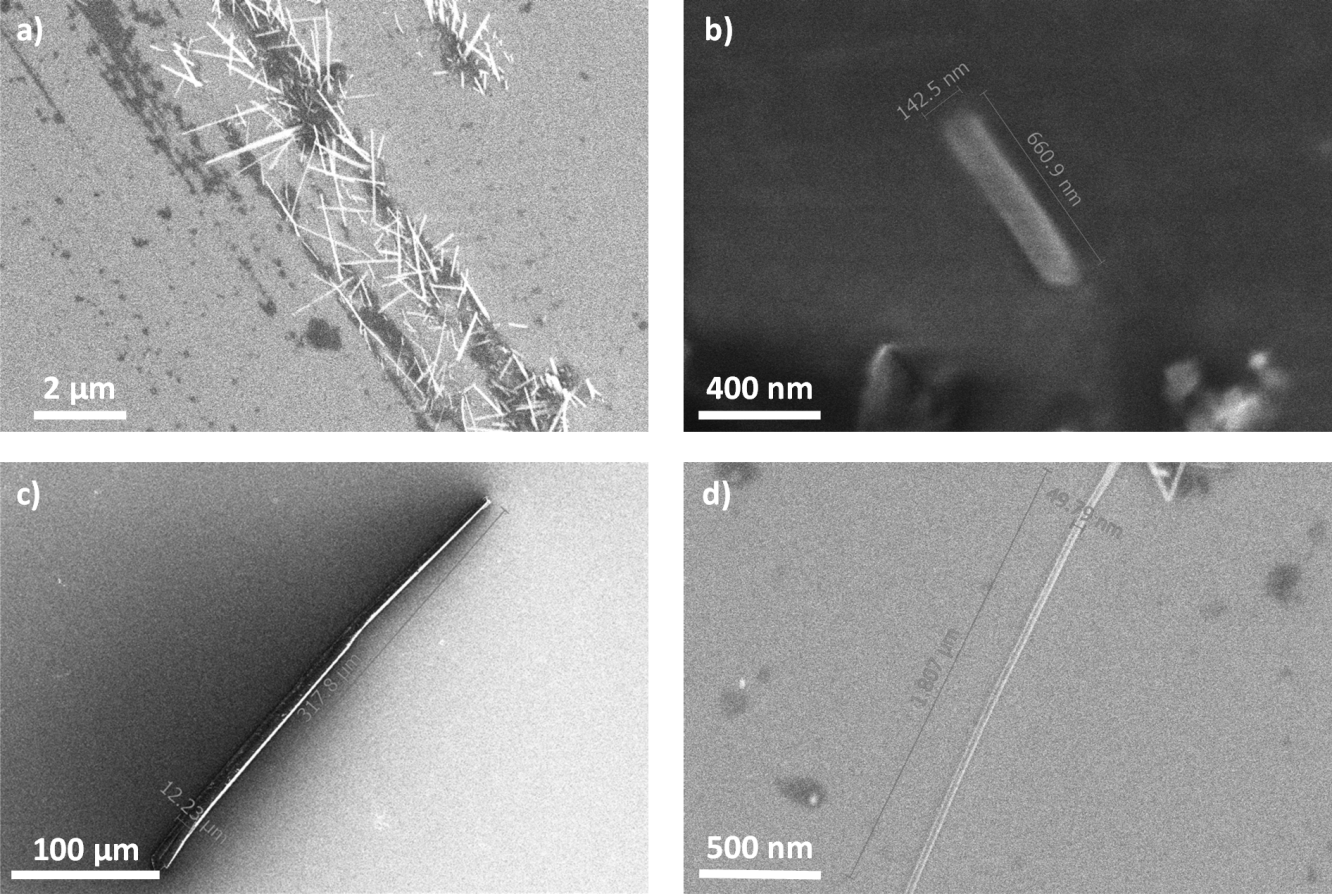
SEM images of BPP-OiPr showing: a) the variety in crystallization, including differences in shape, length, and width, b) rod-like crystals with lengths of hundreds of nanometers, c) rod-like crystals with lengths of hundreds of micrometers, and d) longer wire.

Nano- and microcrystals of organic semiconductors exhibit great potential in next-generation nanoscale optoelectronics and photonics [32–35]. However, precise preparation and shape control over organic crystals are still elusive targets [36]. We carried out SEM analysis of crystals of BPP-OiPr **3** obtained by slow evaporation of its solution in a mixture of dichloromethane and *n*-hexane (Figure 3). The formation of rod-shaped nano- and microcrystals and longer wires were revealed, with the widths from tens of nanometers to tens of micrometers and the lengths from hundreds of nanometers to hundreds of micrometers. For example, a nanocrystal (width: 143 nm, length: 661 nm; Figure 3b) and microcrystal (width: 12 µm, length: 318 µm; Figure 3c) of BPP-OiPr **3** were observed along with a long nanowire with the width of ≈50 nm and length over 1.8 µm (Figure 3d). Moreover, nano- and microcrystals of BPP **2** and BPP-dione **4** with similar shapes were also obtained and visualized by SEM (Figures S6 and S7 in Supporting Information File 1), suggesting that the BPP core can lead to such rod-shaped crystals and nanowires.

## Conclusion

In summary, we achieved a facile synthesis of BPP-OiPr **3** and studied its optical properties in comparison to pristine BPP **2** and its oxidation product BPP-dione **4**. Both BPP-OiPr **3** and BPP-dione **4** displayed significantly enhanced PLQYs compared to BPP **2**, and only **4** displayed the intramolecular charge-transfer character. Additionally, these BPPs formed rod-shaped nano- and microcrystals as well as elongated nanowires with the lengths from hundreds of nanometers to hundreds of micrometers, as demonstrated by SEM. These results provide not only an easy access to highly emissive BPP derivatives with potential as organic fluorescent materials, but also an insight to design derivatives of other PAHs with enhanced fluorescence and charge transfer character.

## Supporting Information

File 1 Experimental and computational details, X-ray crystallography, synthesis and characterization of new compounds, additional PL, mass, and NMR spectra, and theoretical calculations.

## Data Availability

Data generated and analyzed during this study is available from the corresponding author upon reasonable request.
