# Supplementary material for: Synthesis and characterizations of highly luminescent 5-isopropoxybenzo[rst]pentaphene
Source: Beilstein J Org Chem. 2025 Feb 4;21:270–6. doi: 10.3762/bjoc.21.19 (PMC11809583; doi:10.3762/bjoc.21.19)
Supplement: File 1 — Experimental and computational details, X-ray crystallography, synthesis and characterization of new compounds, additional PL, mass, and NMR spectra, and theoretical calculations. [file Beilstein_J_Org_Chem-21-270-s001.pdf]

BEILSTEIN JOURNAL OF ORGANIC CHEMISTRY

Supporting Information

for

Synthesis and characterizations of highly luminescent 5-isopropoxybenzo[*rst*]pentaphene

Islam S. Marae, Jingyun Tan, Rengo Yoshioka, Zakaria Ziadi, Eugene Khaskin, Serhii Vasylevskyi, Ryota Kabe, Xiushang Xu and Akimitsu Narita

Beilstein J. Org. Chem. **2025,** *21,* 270–276. doi:10.3762/bjoc.21.19

Experimental and computational details, X-ray crystallography, synthesis and characterization of new compounds, additional PL, mass, and NMR spectra, and theoretical calculations

License and Terms: This is a supporting information file under the terms of the Creative Commons Attribution License (https://creativecommons.org/licenses/by/4.0). Please note that the reuse, redistribution and reproduction in particular requires that the author(s) and source are credited and that individual graphics may be subject to special legal provisions.
The license is subject to the *Beilstein Journal of Organic Chemistry* terms and conditions: (https://www.beilstein-journals.org/bjoc/terms)

1. General experimental details

All reactions when working with air- or moisture-sensitive compounds were carried out under an argon atmosphere using standard Schlenk line techniques. All starting materials and reagents were purchased from commercial sources and used as received unless otherwise noted. The synthesis of 2,2'-(naphthalene-1,4-diyl)dibenzaldehyde (**1**) was performed according to a previously reported procedure.[1] Anhydrous *N,N*-dimethylformamide (DMF), tetrahydrofuran (THF), and toluene were purified by a solvent purification system (GlassContour) prior to use. Thin-layer chromatography (TLC) was done on silica gel sheets with F254 indicator and column chromatography separation was performed with silica gel (particle size 0.063–0.200 mm). Melting points were determined on a YAMATO hot stage apparatus (Model MP-21) and were uncorrected. Infrared spectroscopy was recorded on a Bruker VERTEX 80V FTIR spectrometer. Nuclear Magnetic Resonance (NMR) spectra were recorded in $CDCl_3$ using a Bruker Avance Neo 500 MHz NMR spectrometer. Chemical shifts (δ) were expressed in ppm relative to the signals of the residual solvent ($CDCl_3$, ^{1}H: 7.26 ppm, ^{13}C: 76.00 ppm). Coupling constants (*J*) were recorded in hertz. Abbreviations: s = singlet, d = doublet, t = triplet, m = multiplet. Mass spectrometry (MS) analyses were performed with Bruker timsTOF spectrometer by atmospheric pressure chemical ionization (APCI), including high-resolution (HR) measurements.

UV–vis absorption spectra and photoluminescence spectra were recorded on a HORIBA Duetta at room temperature using a 10 mm quartz cell. Photoluminescence quantum yields (PLQY) were measured using an integrating sphere with a photoluminescence measurement unit (Quantaurus-QY, C11347-01, Hamamatsu Photonics). PLQY measurement for BPP-OiPr **3** and BPP-dione **4** was carried out in toluene, THF, dichloromethane (CH_2Cl_2), and DMF (10^{-5} mol/L) at excitation wavelength of 380 nm (for BPP-OiPr **3**) and 470 nm (for BPP-dione **4**).

2. Synthetic details

5-Isopropyloxybenzo[*rst*]pentaphene (**3**)

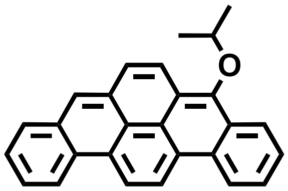

A 1 L flask was charged with 2,2'-(naphthalene-1,4-diyl)dibenzaldehyde (**1**) (1.0 g, 3.0 mmol) and CH_2Cl_2 (500 mL). To this solution, a solution of stannous chloride dihydrate ($SnCl_2·2H_2O$, 20 g, 90 mmol) in isopropanol (iPrOH, 90 mL) was added. To the reaction mixture, concentrated H_2SO_4 (39 mL) was added dropwise at 0 °C under an argon atmosphere. The reaction mixture was allowed to warm to room temperature and stirred for 48 hours in the dark. Then, the reaction was quenched by adding water

(500 mL). The separated organic phase was washed with brine, dried over $MgSO_4$, and concentrated. The residue was purified by silica gel column chromatography (eluent: hexane) to give the title compound (590 mg, 55%) as light yellow solid. ^1H NMR (500 MHz, $CDCl_3$, 298 K) δ 9.18 – 9.12 (m, 2H), 9.06 – 9.01 (m, 2H), 8.55 (d, J = 7.7 Hz, 1H), 8.27 (s, 1H), 8.20 (d, J = 8 Hz, 1H), 8.16 (d, J = 9.5 Hz, 1H), 7.86 – 7.74 (m, 5H), 4.76 – 4.68 (m, 1H), 1.51 (s, 6H). ^{13}C NMR (126 MHz, $CDCl_3$, 293 K) δ 148.45, 131.69, 130.35, 129.75, 128.68, 128.65, 128.41, 127.85, 127.64, 126.5, 126.38, 126.20, 126.04, 125.53, 125.17, 124.75, 124.26, 123.74, 123.24, 123.20, 123.10, 122.29, 122.17, 121.13, 78.12, 22.93. HR MS (APCI): m/z Calcd. For $C_{27}H_{21}O^+$: 361.1587, $[M+H]^+$, found: 361.1583; FTIR (powder) 3073, 3038, 2965, 2919, 1623, 1553, 1466, 1370, 1335, 1303, 1241, 1171, 1086, 1017, 867, 727 cm^{-1}; melting point (m.p.): 155-157 °C; TLC R_f (hexane): 0.1.

Benzo[*rst*]pentaphene-5,8-dione (**4**)[2]

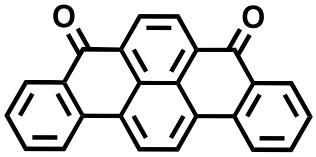

A flask was charged with 5-isopropyloxybenzo[*rst*]pentaphene (**3**) (0.25 g, 0.69 mmol), SiO_2 (1.2 g, 20 mmol), anhydrous $FeCl_3$ (1.1 g, 6.8 mmol), and CH_2Cl_2 (100 mL). The reaction mixture was refluxed at 45 °C under air for 5 days. After cooling to room temperature, the reaction mixture was poured into 200 mL of water and then extracted with CH_2Cl_2 (100 mL) five times. The separated organic phases were combined, washed with brine, dried over $MgSO_4$, filtered and concentrated. The residue was washed with CH_2Cl_2 and filtered to give the title compound (140 mg, 61%) as brown solid. ^1H NMR (500 MHz, $CDCl_3$, 298 K) δ 8.92 (s, 2H), 8.55 (s, 2H), 8.49 (d, J = 7.9 Hz, 2H), 8.35 (d, J = 7.9 Hz, 2H), 7.86 – 7.74 (m, 2H), 7.60 (t, J = 7.5 Hz, 2H).^{13}C NMR (126 MHz, $CDCl_3$, 298 K) δ 183.65, 135.78, 134.09, 132.38, 130.66, 129.21, 129.16, 128.99, 128.55, 128.37, 124.43, 123.37. HR MS (APCI): m/z Calcd. For $C_{24}H_{13}O_2^+$: 333.0910, $[M+H]^+$, found: 333.0881. FTIR (powder) 1645, 1592, 1569, 1452, 1371, 1289, 1274, 1143, 930, 843, 755, 691, 637 cm^{-1}; m.p.: over 250 °C;

3. X-ray single crystallography

A suitable light-orange, needle-shaped crystal of BPP-OiPr **3** was selected and mounted on a Bruker SMART APEX3 area detector diffractometer.[3] The crystal was kept at a steady T = 100 K during data collection. Data were measured using ω,φ-scans with Cu radiation. The unit cell was refined using SAINT V8.40B (Bruker, 2016) on 23325 reflections with $C222_1$ space group. Data reduction, scaling, and absorption corrections were performed using SAINT V8.40B (Bruker, 2016/2). The structure was solved with the ShelXT 2018/2 (Sheldrick, 2018) solution program using iterative methods and by using Olex2 1.5 as a graphical interface[4]. The model was refined

with ShelXL 2016/6 using full matrix least squares minimization on F^2.[5] All non-hydrogen atoms were refined anisotropically. Hydrogen atom positions were calculated geometrically and refined using the riding model. _exptl_absorpt_process_details: SADABS-2016/2 (Bruker,2016/2) was used for absorption correction.[6] The structure was deposited at the Cambridge Crystallographic Data Centre (CCDC) and the data can be obtained free of charge via www.ccdc.cam.ac.uk/structures. Crystal data for compound BPP-O*i*Pr **3** (CCDC number: 2372328).

Table S1. Crystal data and structure refinement for BPP-OiPr **3**

Compound	BPP-OiPr **3**
Identification code	2372328
Empirical formula	$C_{27}H_{20}O$
Formula weight	$C_{27}H_{20}O$
Temperature/K	293
Space group	*P*-1
a/Å	8.8843(1)
b/Å	13.4942(2)
c/Å	15.9674(3)
α/°	104.597(1)
β/°	104.978(1)
γ/°	101.030(1)
Volume/Å^3	1720.89(5)
Z	4
ρ_{calc}g/cm^3	1.391
μ/mm^{-1}	0.638
F(000)	760.0
Radiation	CuKα (λ = 1.54184)
wR_2 (all data)	0.1687
R(reflections)	0.0595

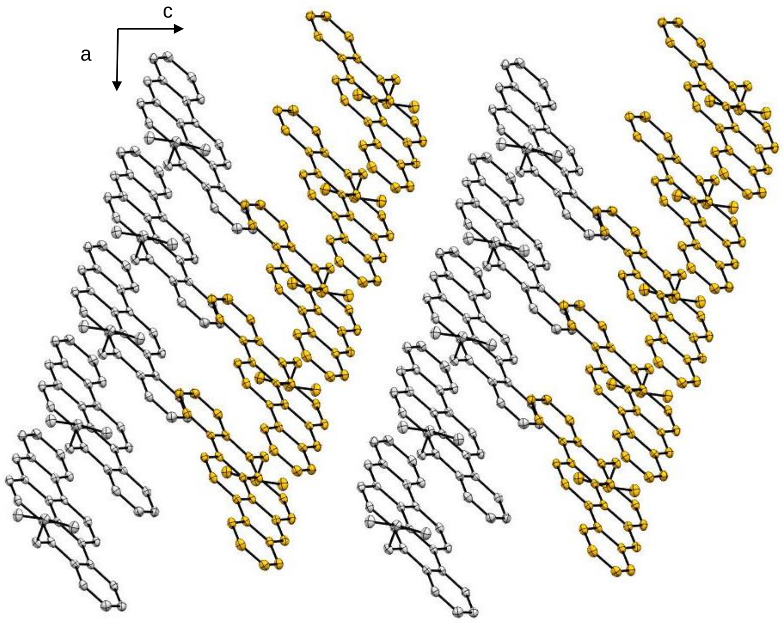

Figure S1. Packing structure of BPP-OiPr **3** using thermal ellipsoids model.

4. DFT calculations

DFT calculations were performed using the Gaussian 09 software package.[7] The geometries, molecular orbitals, and MO energy were calculated at B3LYP/6-311G(d,p) level of theory[8, 9]. Time-dependent DFT (TD-DFT) calculations were performed with M062X/6-311G(d,p)[10] using toluene as the solvent model (SMD)[11].

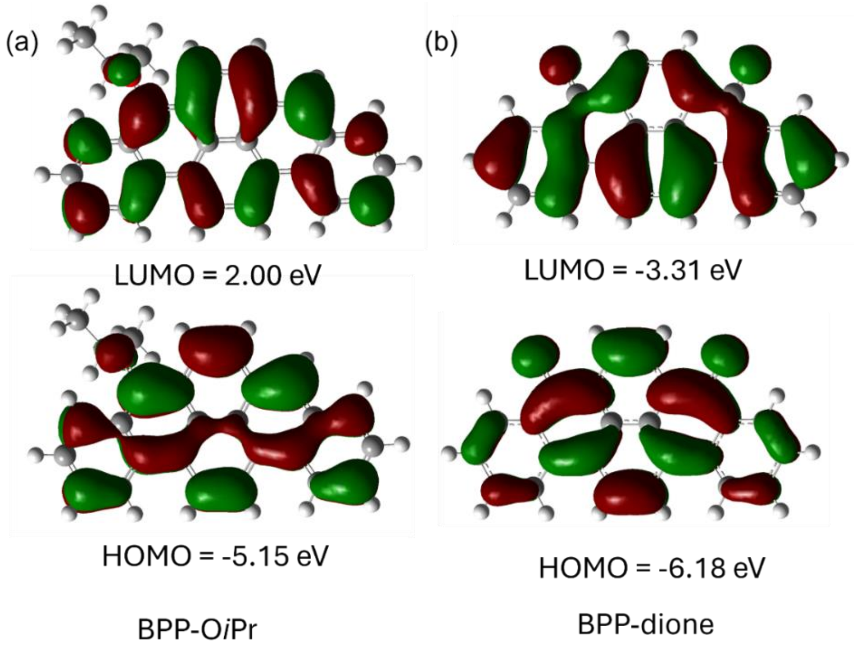

Figure S2. Molecular orbitals and HOMO and LUMO energy diagrams of (a) BPP-OiPr **3** and (b) BPP-dione **4**.

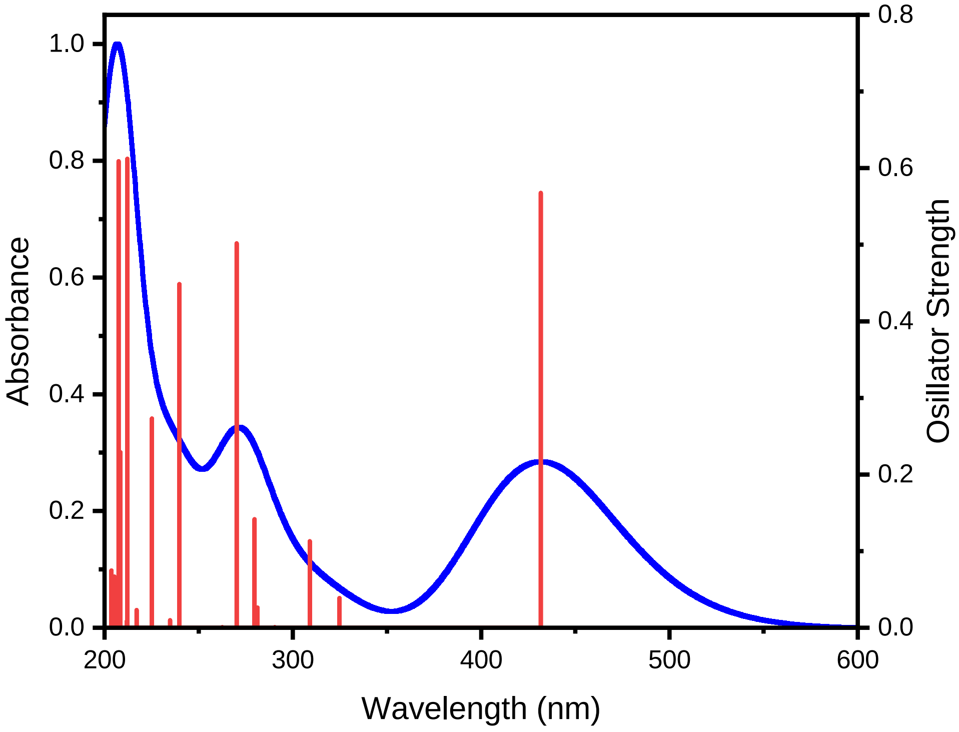

Figure S3. Simulated absorption spectrum of BPP-dione **4** (blue curve) and the oscillator strengths (red bars) from TDDFT calculations at the M062X/6-311G(d,p) level using toluene as the solvent (model: SMD).

Table S2. The representative transitions of BPP-OiPr **3** (S1-S10) calculated by TD-DFT at the M06-2X/6-311G(d,p) level using toluene as the solvent

Excited states	Transition energy (eV)	Wavelength (nm)	Oscillator strength	Description
S1	3.2216	384.86	1.0601	HOMO → LUMO (0.69557)
S2	3.4440	360.00	0.0257	HOMO-1 → LUMO (0.31691)<br>HOMO → LUMO+1 (0.60390)
S3	4.1161	301.22	0.2163	HOMO-1 → LUMO (0.59324)<br>HOMO → LUMO+1 (0.34011)<br>HOMO → LUMO+2 (0.15283)
S4	4.4319	279.76	0.0137	HOMO-1→ LUMO+1 (0.11070)<br>HOMO → LUMO+2 (0.21189)<br>HOMO → LUMO+3 (0.56062)<br>HOMO → LUMO+4 (0.31388)
S5	4.5626	271.74	1.3622	HOMO-1 → LUMO+1 (0.66406)<br>HOMO → LUMO (0.10179)<br>HOMO → LUMO+3 (0.13816)
S6	4.6554	266.32	0.0665	HOMO-3 → LUMO (0.36997)

				HOMO-2 → LUMO+1 (0.31712)<br>HOMO-1 → LUMO+1 (0.10318)<br>HOMO-1 → LUMO+2 (0.26006)<br>HOMO → LUMO+3 (0.21263)<br>HOMO → LUMO+4 (0.31467)
S7	4.7156	262.92	0.1378	HOMO-3 → LUMO+1 (0.10221)<br>HOMO-1 → LUMO (0.17228)<br>HOMO → LUMO+2 (0.61673)<br>HOMO → LUMO+3 (0.21381)
S8	4.9143	252.29	0.1562	HOMO-2 → LUMO (0.66076)<br>HOMO-1 → LUMO+4 (0.10665)
S9	5.0051	247.72	0.0209	HOMO-4 → LUMO (0.35675)<br>HOMO-3 → LUMO (0.49888)<br>HOMO-2 → LUMO+1 (0.14923)<br>HOMO → LUMO+3 (0.18402)<br>HOMO → LUMO+4 (0.20158)
S10	5.2528	236.03	0.0188	HOMO-4 → LUMO (0.11858)<br>HOMO-4 → LUMO+1 (0.19320)<br>HOMO-3 → LUMO+1 (0.28488)<br>HOMO-1 → LUMO+2 (0.24083)<br>HOMO-1 → LUMO+3 (0.45007)

Table S3. The representative transitions of BPP-dione **4** (S1-S10) calculated by TD-DFT at the M06-2X/6-311G(d,p) level using toluene as the solvent

Excited states	Transition energy (eV)	Wavelength (nm)	Oscillator strength	Description
S1	2.8720	431.69	0.5674	HOMO → LUMO (0.70084)
S2	3.2788	378.14	0.0000	HOMO-7 → LUMO+1 (0.26514)<br>HOMO-7 → LUMO+3 (0.10079)<br>HOMO-7 → LUMO+7 (0.12882)<br>HOMO-6 → LUMO (0.59314)<br>HOMO-6 → LUMO+2 (0.15271)
S3	3.3092	374.67	0.0000	HOMO-7 → LUMO (0.58657)<br>HOMO-7 → LUMO+2 (0.16174)<br>HOMO-6 → LUMO+1 (0.26941)<br>HOMO-6 → LUMO+3 (0.10150)<br>HOMO-6 → LUMO+7 (0.12845)
S4	3.8179	324.74	0.0387	HOMO-5 → LUMO (0.16822)<br>HOMO-3 → LUMO (0.23437)<br>HOMO-1 → LUMO (0.60246)<br>HOMO → LUMO+3 (0.17606)
S5	4.0125	309.00	0.1128	HOMO-4 → LUMO (0.21995)

				HOMO-1 → LUMO (0.64053)<br>HOMO → LUMO+2 (0.12510)
S6	4.2694	290.40	0.0007	HOMO-3 → LUMO (0.49081)<br>HOMO-1 → LUMO (0.12996)<br>HOMO → LUMO+1 (0.43617)
S7	4.4093	281.19	0.0262	HOMO-4 → LUMO (0.58443)<br>HOMO-3 → LUMO+1 (0.14396)<br>HOMO-2 → LUMO (0.13921)<br>HOMO → LUMO+2 (0.27631)
S8	4.4339	279.63	0.1415	HOMO-5 → LUMO (0.56519)<br>HOMO-4 → LUMO+1 (0.11549)<br>HOMO-3 → LUMO (0.11064)<br>HOMO-1 → LUMO (0.26414)<br>HOMO → LUMO+1 (0.12164)<br>HOMO → LUMO+3 (0.18227)<br>HOMO → LUMO+5 (0.11495)
S9	4.5891	270.17	0.5016	HOMO-4 → LUMO (0.23225)<br>HOMO-2 → LUMO (0.20113)<br>HOMO → LUMO+2 (0.62086)
S10	4.7228	262.52	0.0007	HOMO-5 → LUMO (0.22825)<br>HOMO-3 → LUMO (0.39485)<br>HOMO-2 → LUMO (0.12365)<br>HOMO → LUMO+1 (0.50260)

Table S4. Cartesian coordinates of the DFT-optimized BPP-OiPr **3** at the B3LYP/6-311G(d,p) level.

Symbol	X	Y	Z
C	-0.38317	1.527348	0.031597
C	0.001638	0.179675	-0.16448
C	1.385154	-0.18638	-0.13955
C	2.385856	0.7917	0.085355
C	1.963169	2.131723	0.290651
C	0.641795	2.481936	0.26351
C	-0.99974	-0.84067	-0.36217
C	-0.57717	-2.20267	-0.58062
C	0.730025	-2.54094	-0.57746
C	1.764264	-1.56281	-0.34798
C	3.780778	0.40634	0.103219
C	-1.78186	1.886857	-0.03637
C	4.122637	-0.96422	-0.10948
C	5.486991	-1.35303	-0.09671
C	6.48462	-0.43316	0.117263
C	6.152665	0.920419	0.32796
C	4.835919	1.325205	0.320492

C	-2.23556	3.223239	0.079868
C	-3.56893	3.546887	-0.03576
C	-4.52176	2.54278	-0.29293
C	-4.12179	1.232154	-0.40016
C	-2.7588	0.867779	-0.25112
C	3.095948	-1.91481	-0.33042
C	-2.34171	-0.49488	-0.35717
O	-3.28849	-1.47538	-0.53921
C	-4.08362	-1.86965	0.618908
C	-5.27421	-2.62956	0.055286
C	-3.26448	-2.69666	1.604026
H	2.696521	2.90587	0.471992
H	0.383321	3.519486	0.427415
H	-1.34295	-2.94227	-0.76802
H	1.031407	-3.5681	-0.75447
H	5.72899	-2.39786	-0.26105
H	7.523382	-0.74343	0.124159
H	6.938481	1.648058	0.496471
H	4.615624	2.371747	0.484848
H	-1.52309	4.019206	0.24985
H	-3.88274	4.58063	0.05465
H	-5.56632	2.805115	-0.41691
H	-4.84224	0.459676	-0.63222
H	3.373534	-2.95192	-0.49109
H	-4.42874	-0.9587	1.119805
H	-5.93667	-2.95397	0.861699
H	-5.84554	-2.00431	-0.63388
H	-4.93298	-3.51351	-0.4897
H	-3.87264	-2.93273	2.482042
H	-2.38203	-2.15119	1.943384
H	-2.93934	-3.63633	1.150225

Table S5. Cartesian coordinates of the DFT-optimized BPP-dione **4** at the B3LYP/6-311G(d,p) level.

Symbol	X	Y	Z
C	-5.05867	-2.10903	0.000932
C	-3.6722	-2.14458	0.000857
C	-2.9119	-0.96155	0.000126
C	-3.61066	0.264946	-0.00027
C	-5.01068	0.290261	-0.00023
C	-5.7381	-0.88779	0.000319
C	-1.43756	-0.9723	-0.00018
C	-0.71585	0.26223	6.78E-05
C	-1.40098	1.506909	-4.2E-05
C	-2.8895	1.561447	-0.0005
C	-0.70001	-2.14543	-0.00084
C	0.700014	-2.14543	-0.00083
C	1.437559	-0.9723	-9.7E-05

C	0.715844	0.262231	0.000219
C	1.400977	1.506911	0.000485
C	0.697294	2.701161	0.000412
C	-0.6973	2.70116	9.23E-05
C	2.911899	-0.96155	3.58E-05
C	3.610662	0.264947	0.000291
C	2.889502	1.56145	0.000945
C	3.672206	-2.14458	-0.00014
C	5.058668	-2.10902	-0.00023
C	5.738099	-0.88779	-0.00012
C	5.010681	0.290262	0.000174
O	-3.48424	2.629482	-0.00111
O	3.484242	2.629486	9.66E-06
H	-5.61532	-3.03948	0.001524
H	-3.1826	-3.10926	0.001522
H	-5.49652	1.258012	-0.00058
H	-6.82146	-0.86398	0.000343
H	-1.19999	-3.10465	-0.00154
H	1.19999	-3.10465	-0.00149
H	1.261018	3.62583	0.000619
H	-1.26102	3.625828	-7.7E-05
H	3.182605	-3.10926	-0.00018
H	5.615325	-3.03948	-0.00038
H	6.821466	-0.86398	-0.00021
H	5.49652	1.258012	0.000387

5. Photophysical properties

Table S6. Summary of photophysical data of BPP **2**, BPP-OiPr **3**, and BPP-dione **4**.

	λ_{abs}[a] [nm]	λ_{em}[b] [nm]	Φ_{PL}[c] (toluene/CH_2Cl_2/THF/DMF)	HOMO/ LUMO[d] [eV]	E_{gap}[e] [eV]
BPP **2**	360, 383, 416	436, 463, 494	0.13/--/--/--	–5.30/ –2.06	3.24
BPP-O*i*Pr **3**	375, 398, 442	444, 472, 504	0.73/0.4/--/0.67	−5.15/ −2.00	3.15
BPP-dione **4**	305, 469, 497	538, 572	0.62/0.72/0.70/0.69	−6.18/ −3.31	2.87

[a]Measured in 10^{-5} M toluene solution. [b]Measured in 10^{-5} M toluene solution with excitation at 360 nm. [c]Absolute photoluminescence quantum yield measured in different solvents. [d]HOMO/LUMO energy levels were calculated by DFT at the B3LYP/6-311G(d,p) level. [e]Energy gap (E_{gap}) was calculated according to E_{gap} = LUMO – HOMO.

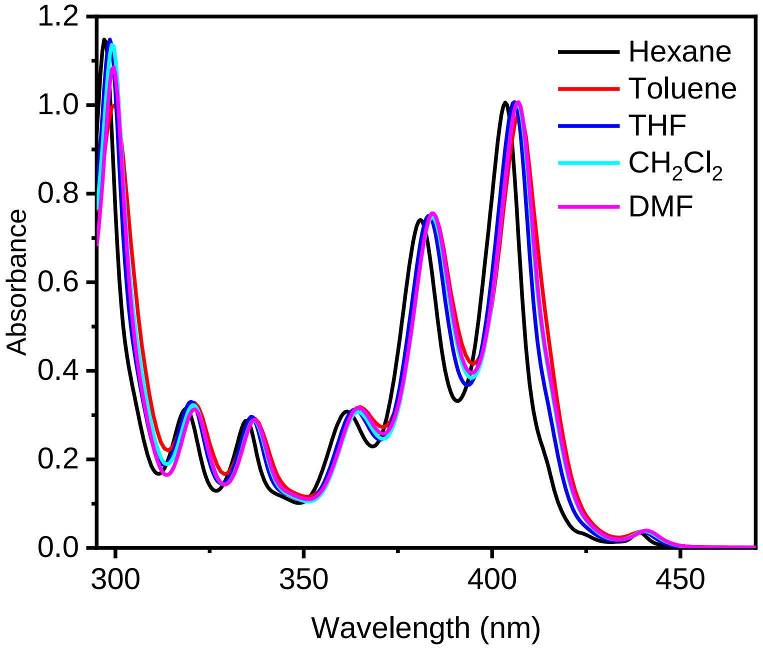

Figure S4. Normalized absorption spectra of BPP-OiPr **3** (10^{-5} mol/L) measured in hexane, toluene, tetrahydrofuran (THF), dichloromethane (CH_2Cl_2), and dimethylformamide (DMF).

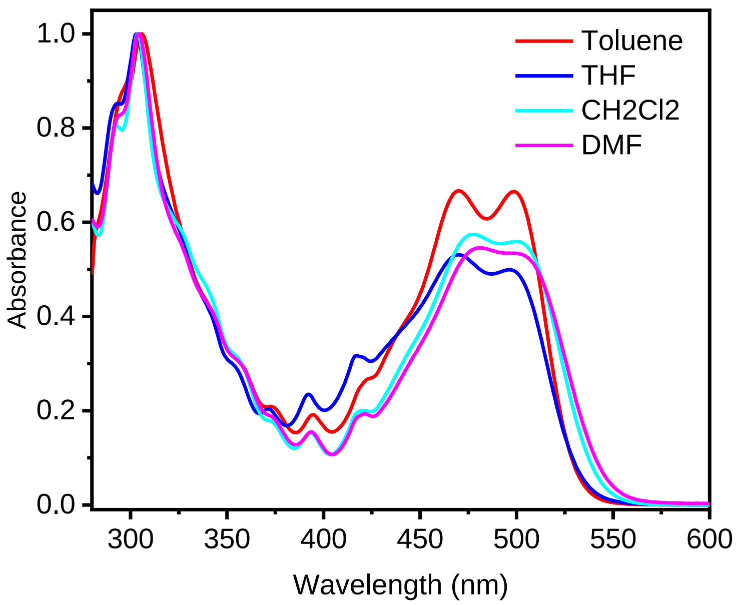

Figure S5. Normalized absorption spectra of BPP-dione **4** (10^{-5} mol/L) measured in toluene, tetrahydrofuran (THF), dichloromethane (CH_2Cl_2), and dimethylformamide (DMF).

6. Scanning electron microscopy (SEM)

SEM measurements were performed using an FEI Helios G3 UC dual-beam microscope. After the crystals of BPP **2** and BPP-dione **4** being obtained by slow evaporation of dichloromethane, they were placed on clean Si substrates, which were sonicated in acetone and isopropanol prior to usage. A drop of isopropanol was added on top to disperse the BPP crystals and ensure they were firmly attached to the surface once the isopropanol dried under atmospheric conditions. Images were acquired using an accelerating voltage of 5 kV, a beam current of 13 pA, and a working distance of 4 mm. Secondary electron imaging mode was employed to capture high-resolution surface topography. Multiple areas of each sample were imaged to ensure representative results.

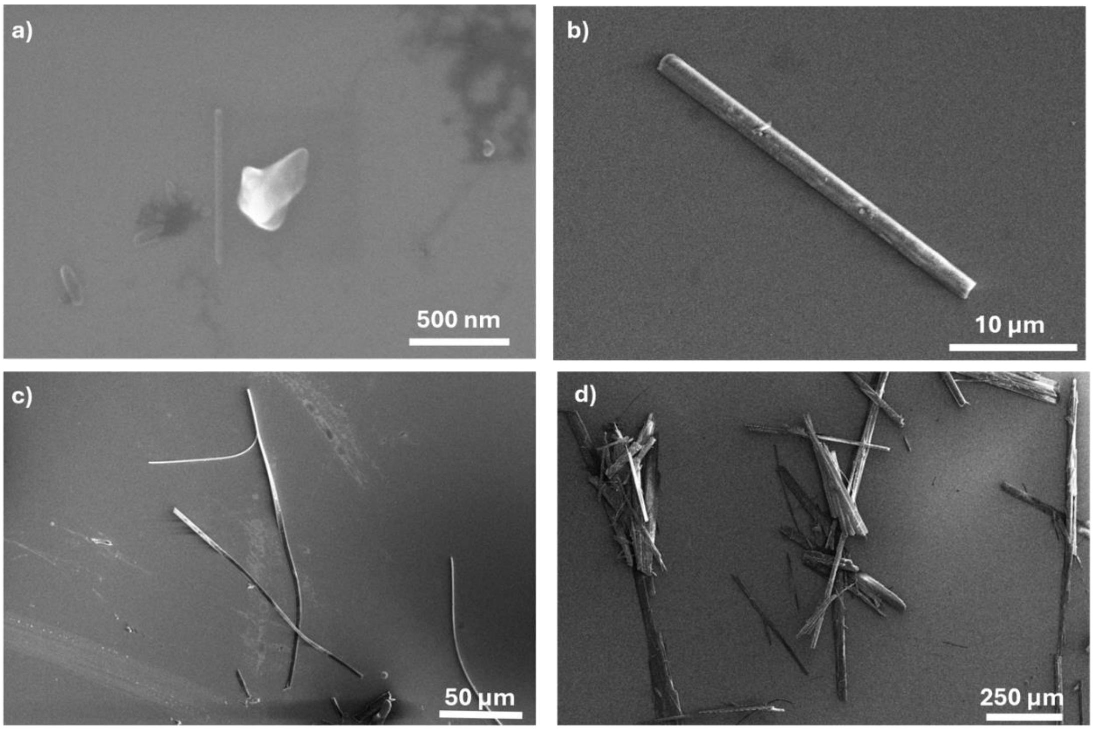

Figure S6. SEM images of BPP **2** showing similar crystallization patern as BPP-OiPr **3** as in: (a) rod-like crystals with lengths of hundreds of nanometers, (b) rod-like crystals with lengths of hundreds of micrometers, (c) long supramolecular wire, and (d) the variety in crystallization, including differences in shape, length, and width.

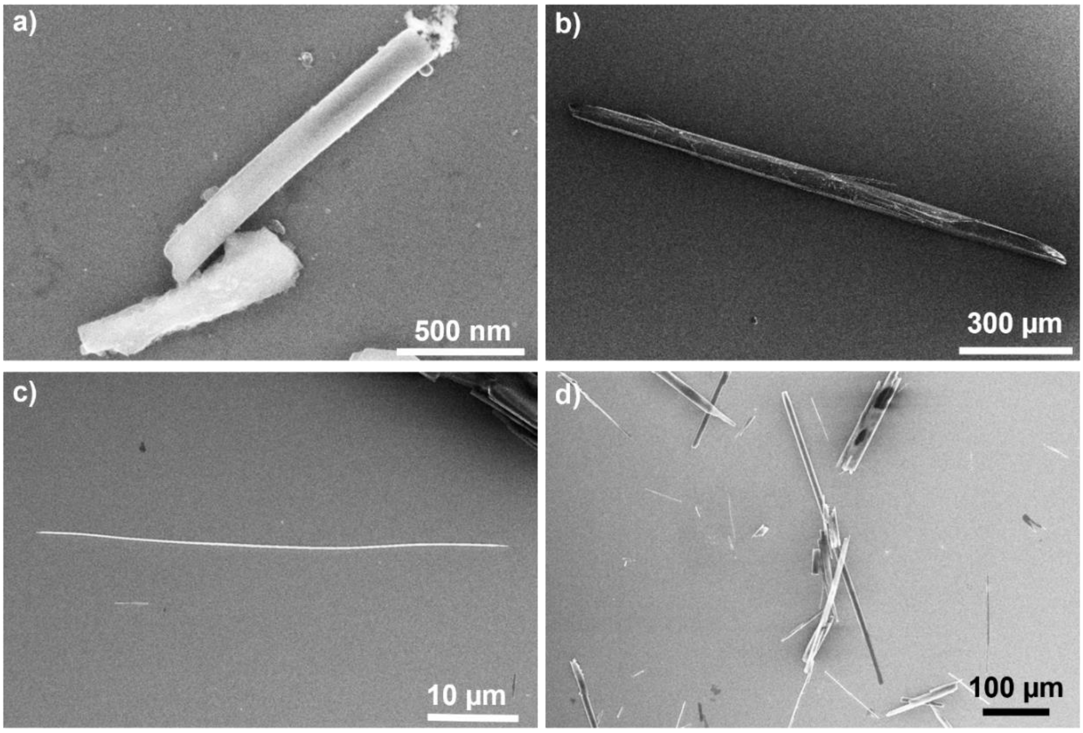

Figure S7. SEM images of BPP-dione **4** showing similar crystallization patern as BPP-OiPr **3** as in: (a) rod-like crystals with lengths of hundreds of nanometers, (b) rod-like crystals with lengths of hundreds of micrometers, (c) long wire, and (d) the variety in crystallization, including differences in shape, length, and width.

7. NMR and MS spectra

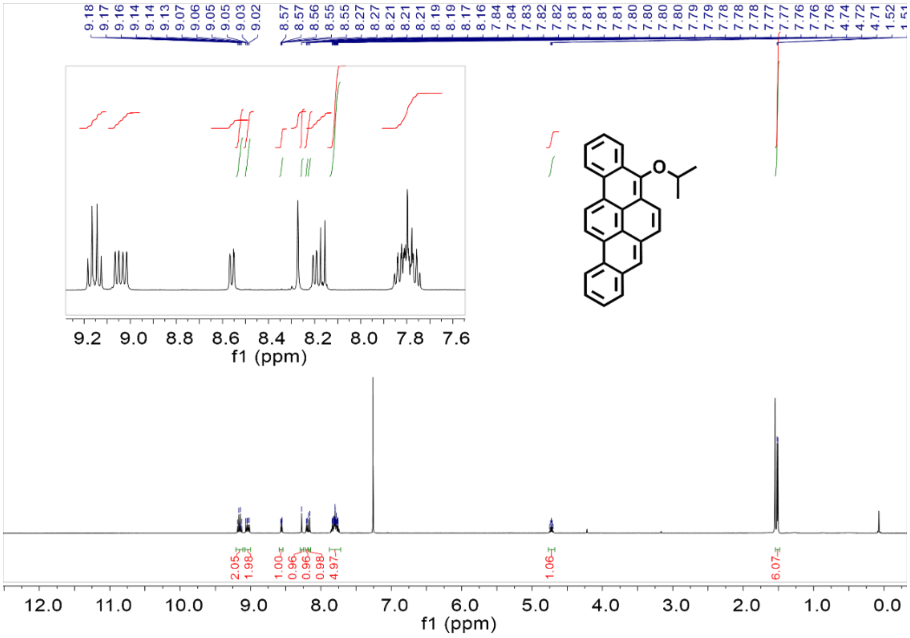

Figure S8. ^{1}H NMR spectrum of compound BPP-OiPr **3** in $CDCl_3$ (500 MHz, 298 K).

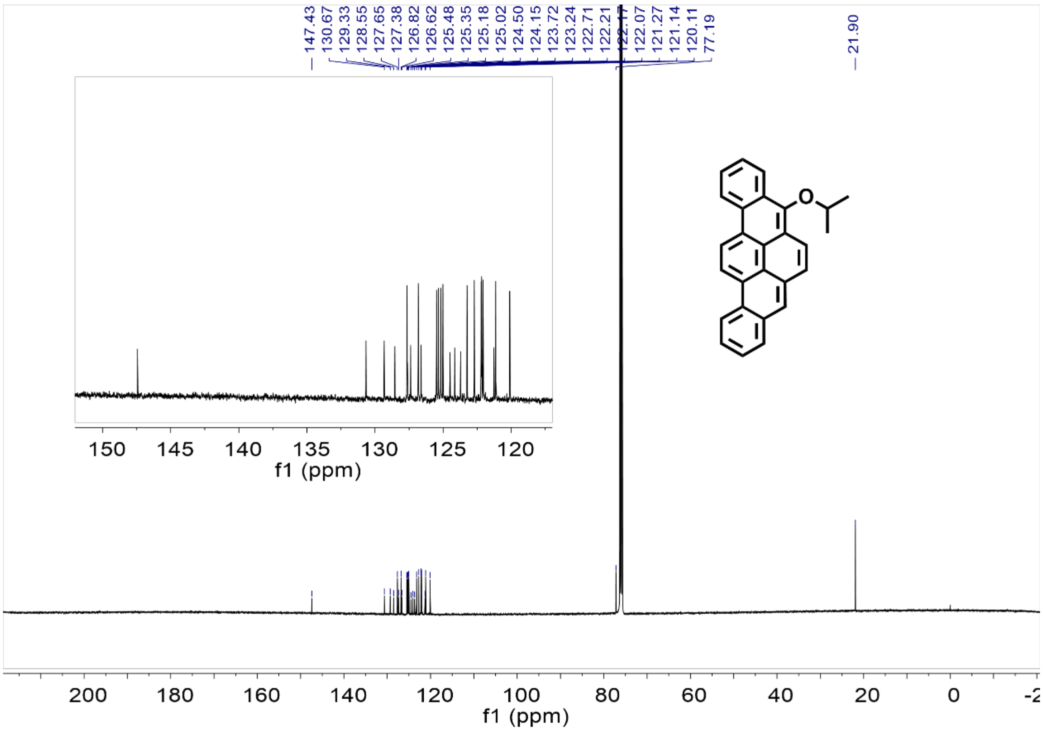

Figure S9. ^{13}C NMR spectrum of compound BPP-OiPr **3** in $CDCl_3$ (126 MHz, 298 K).

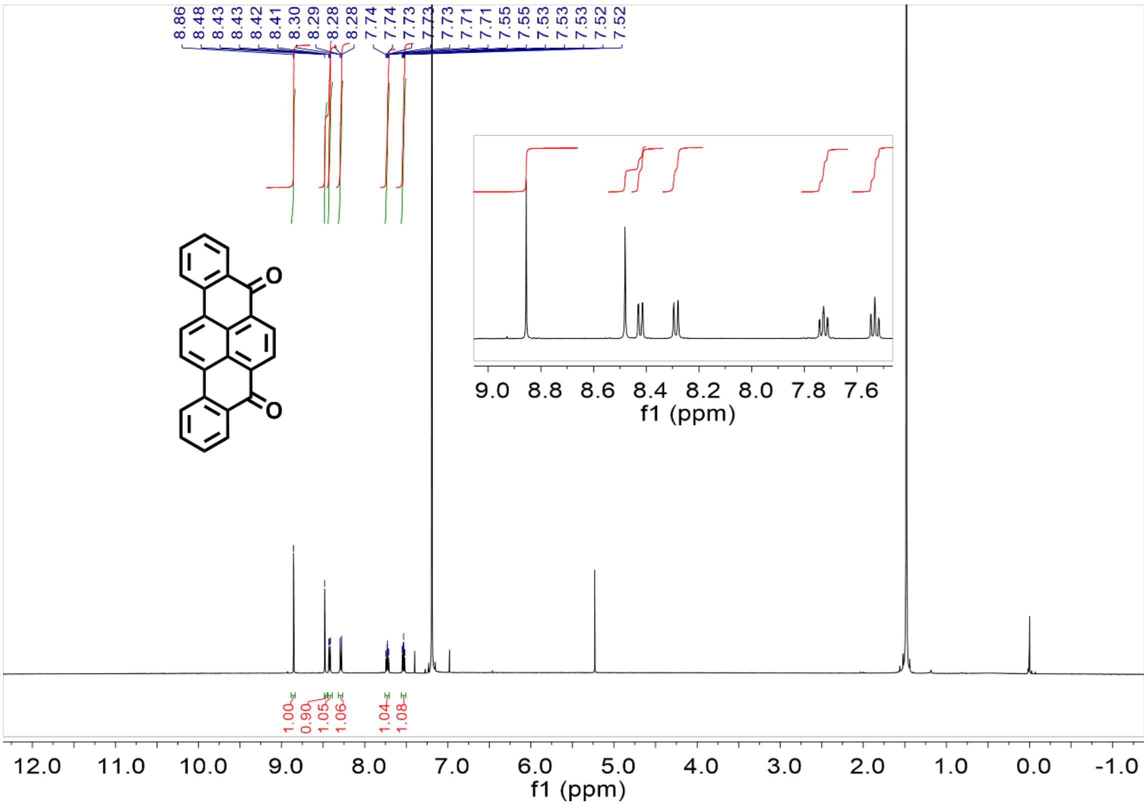

Figure S10. ^{1}H NMR spectrum of compound BPP-dione **4** in $CDCl_3$ (500 MHz, 298 K).

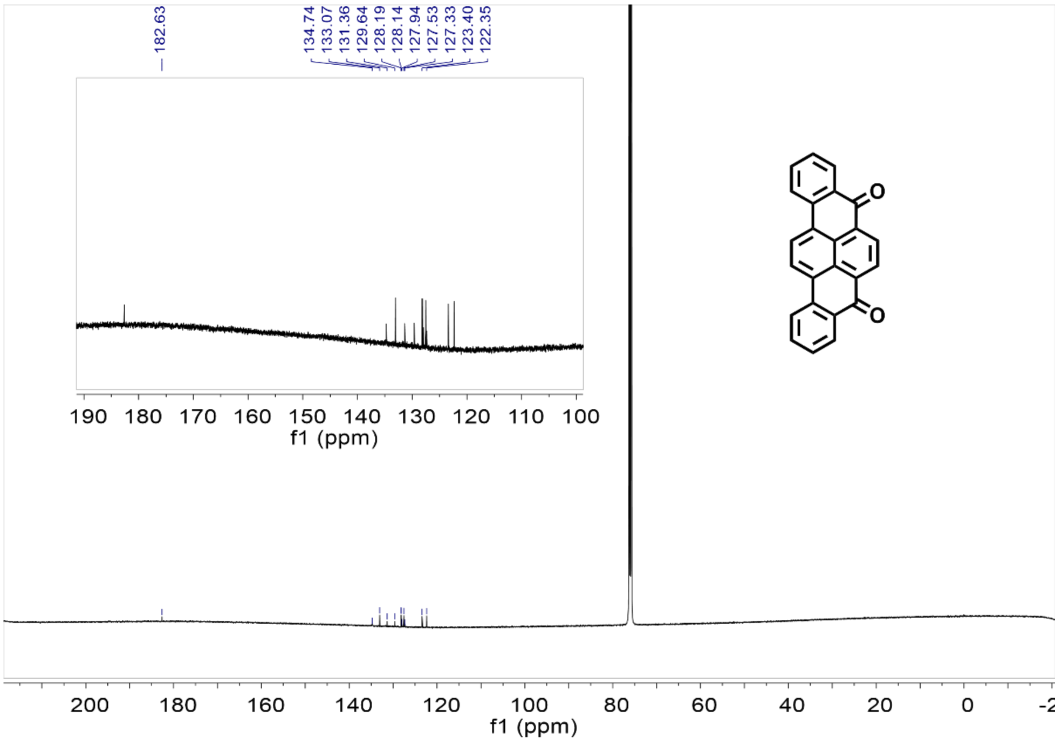

Figure S11. ^{13}C NMR spectrum of compound BPP-dione **4** in $CDCl_3$ (126 MHz, 298 K).

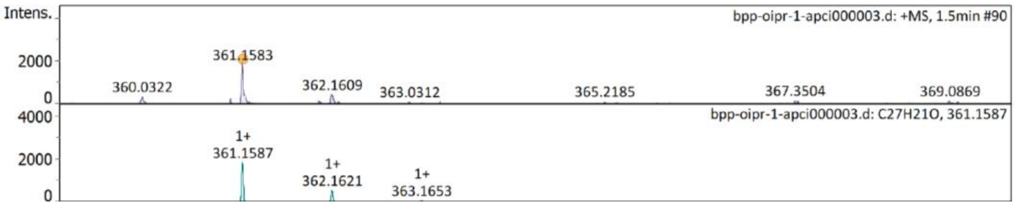

Figure S12. High-resolution APCI MS spectrum of BPP-O*i*Pr **3** (Top: experimental isotopic distribution pattern of BPP-OiPr **3**; down: calculated isotopic distribution pattern of BPP-dione).

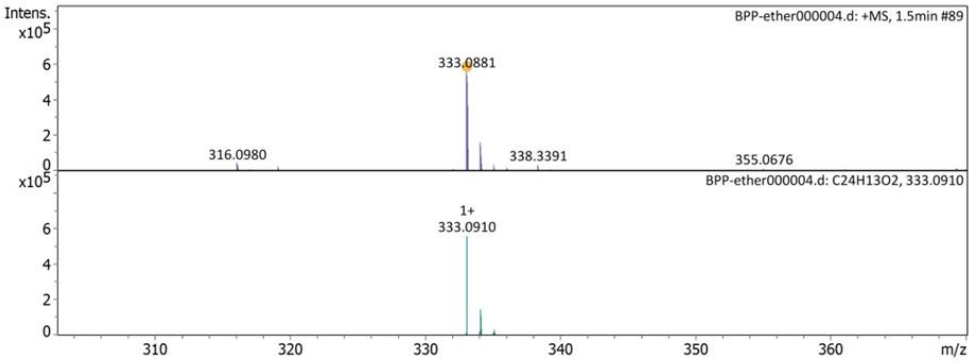

Figure S13. APCI MS spectrum of BPP-dione **4** (Top: experimental isotopic distribution pattern of BPP-dione **4**; down: calculated isotopic distribution pattern of BPP-dione **4**).

8. References.

[1] Lungerich, D.; Papaianina, O.; Feofanov, M.; Liu, J.; Devarajulu, M.; Troyanov, S. I.; Maier, S.; Amsharov, K. Dehydrative π-extension to nanographenes with zig-zag edges *Nat. Commun.* **2018**, *9*, 4756.

[2] ÜNseren, E.; Fieser, L. F. Investigation of the Metabolism of 3,4,9,10-Dibenzpyrene *J. Org. Chem.* **1962**, *27*, 1386-1389.

[3] CrysAlisPro Software System, Rigaku Oxford Diffraction **2024**.

[4] Dolomanov, O. V.; Bourhis, L. J.; Gildea, R. J.; Howard, J. A. K.; Puschmann, H. OLEX2: a complete structure solution, refinement and analysis program *J. Appl. Crystallogr.* **2009**, *42*, 339-341.

[5] Sheldrick, G. Crystal structure refinement with SHELXL *Acta Crystallogr., Sect.C* **2015**, *71*, 3-8.

[6] Sheldrick, G. SHELXT - Integrated space-group and crystal-structure determination *Acta Crystallogr. A* **2015**, *71*, 3-8.

[7] Frisch, M. J. *Gaussian 09, Revision A.1*; Gaussian, Inc., Wallingford, **2009**.

[8] Stephens, P. J.; Devlin, F. J.; Chabalowski, C. F.; Frisch, M. J. Ab Initio Calculation of Vibrational Absorption and Circular Dichroism Spectra Using Density Functional Force Fields *J. Phys. Chem.* **1994**, *98*, 11623-11627.

[9] Riley, K. E.; Op't Holt, B. T.; Merz, K. M. Critical Assessment of the Performance of Density Functional Methods for Several Atomic and Molecular Properties *J. Chem. Theory Comput.* **2007**, *3*, 407-433.

[10] Zhao, Y.; Truhlar, D. G. Density Functional for Spectroscopy: No Long-Range Self-Interaction Error, Good Performance for Rydberg and Charge-Transfer States, and Better Performance on Average than B3LYP for Ground States *J. Phys. Chem. A* **2006**, *110*, 13126-13130.

[11] Marenich, A. V.; Cramer, C. J.; Truhlar, D. G. Universal Solvation Model Based on Solute Electron Density and on a Continuum Model of the Solvent Defined by the Bulk Dielectric Constant and Atomic Surface Tensions *J. Phys. Chem. B* **2009**, *113*, 6378-6396.
